# The Impact of Glucagon-Like Peptide 1 Receptor Agonists on Bone Metabolism and Its Possible Mechanisms in Osteoporosis Treatment

**DOI:** 10.3389/fphar.2021.697442

**Published:** 2021-06-14

**Authors:** Baocheng Xie, Shichun Chen, Yongxiang Xu, Weichao Han, Runkai Hu, Minyi Chen, Yusheng Zhang, Shaobo Ding

**Affiliations:** ^1^ Department of Pharmacy, Affiliated Dongguan Hospital, Southern Medical University, Dongguan, China; ^2^ Department of Pharmacy, The First People’s Hospital of Foshan (The Affiliated Foshan Hospital of Sun Yat-Sen University), Foshan, China

**Keywords:** glucagon-like peptide-1, diabetes mellitus, glucagon-like peptide 1 receptor agonists, osteoporosis, bone resorption

## Abstract

Diabetes mellitus and osteoporosis are closely related and have complex influencing factors. The impact of anti-diabetic drugs on bone metabolism has received more and more attention. Type 2 diabetes mellitus (T2DM) would lead to bone fragility, high risk of fracture, poor bone repair and other bone-related diseases. Furthermore, hypoglycemic drugs used to treat T2DM may have notable detrimental effects on bones. Thus, the clinically therapeutic strategy for T2DM should not only effectively control the patient’s glucose levels, but also minimize the complications of bone metabolism diseases. Glucagon-like peptide-1 receptor agonists (GLP-1RAs) are novel and promising drug for the treatment of T2DM. Some studies have found that GLP-1RAs may play an anti-osteoporotic effect by controlling blood sugar levels, promoting bone formation and inhibiting bone resorption. However, in clinical practice, the specific effects of GLP-1RA on fracture risk and osteoporosis have not been clearly defined and evidenced. This review summarizes the current research findings by which GLP-1RAs treatment of diabetic osteoporosis, postmenopausal osteoporosis and glucocorticoid-induced osteoporosis and describes possible mechanisms, such as GLP-1R/MAPK signaling pathway, GLP-1R/PI3K/AKT signaling pathway and Wnt/β-catenin pathway, that are associated with GLP-1RAs and osteoporosis. The specific role and related mechanisms of GLP-1RAs in the bone metabolism of patients with different types of osteoporosis need to be further explored and clarified.

## Introduction

Diabetes mellitus (DM) is a chronic disease that develops at an alarming rate worldwide (Mohler et al., 2009; Xu et al., 2018). Related complications such as kidney disease, retinopathy, cardiovascular events, neurological disorders, bone loss, and bone fragility would severely reduce the patient’s quality of life (Napoli et al., 2017; Schwartz et al., 2011). DM often leads to the development of osteoporosis, which is one of the serious complications caused by diabetes. Both type T1DM and T2DM are associated with bone abnormalities and increased risk of fractures (Napoli et al., 2017; Schwartz et al., 2011). GLP-1RAs as novel and promising drugs for T2DM, they could stimulate insulin secretion in a glucose-dependent manner, protect β cell function, and suppress glucagon secretion (Lindamood and Taylor, 2015; Aroda, 2018). Currently, GLP-1RAs have been marketed mainly as liraglutide, exendin-4, albiglutide, dulaglutide and semaglutide (Meier, 2012; Sharma et al., 2018). The therapeutic benefit of these drugs in T2DM has raised interest in whether they affect the mechanism of bone metabolism (Mabilleau et al., 2018; Zhang et al., 2018). It has been reported that GLP-1RAs can enhance bone mineral density (BMD), improve bone quality and prevent fractures in diabetic patients and cell and animal experiments further found that GLP-1RAs have excellent potential anti-osteoporosis benefits for postmenopausal osteoporosis, GIOP and senile osteoporosis (Lu et al., 2015; Zhang et al., 2018; Yang et al., 2019; Zhang et al., 2019a; Zhang et al., 2019b). But, the specific role and related mechanisms of GLP-1RAs in the bone metabolism of patients with different types of osteoporosis need to be further explored and clarified. This review summarizes the current research findings by which GLP-1RAs treatment of osteoporosis and describe possible mechanisms of different types of GLP-1RAs on bone metabolism and osteoporosis.

### Biological Functions of Glucagon-Like Peptide-1

Glucagon-like peptide including GLP-1 and GLP-2, are cleaved from proglucagon. The biological function of GLP-1 is mediated by GLP-1R and is highly specific. Their binding first activates cAMP and intracellular calcium - dominated signal transduction pathways. These signaling pathways have different physiological functions from cAMP in glucose-stimulated insulin secretion. GLP-1 increases glucose-dependent insulin secretion and decreases glucagon secretion after meals. Natural GLP-1 has a short half-life and is easily degraded by the dipeptidyl peptidase-4 (DPP-4) enzyme. GLP-1RAs are GLP-1 with prolonged half life to be more resistant to degradation by DPP-4 enzyme. In clinical practice, GLP-1RAs can mimic the biological activity and function of GLP-1 and are widely used in the treatment of diabetic patients. At present, the main GLP-1RAs drugs that have been marketed include liraglutide, exenatide, albiglutide and dulaglutide (Chun and Butts, 2020). Recent studies have found that GLP-1RAs can not only promote insulin secretion and regulate blood sugar, but also affect the body’s bone metabolism through a variety of ways, and play a role in preventing and treating osteoporosis.

### The Effect of Glucagon-Like Peptide-1 Receptor Agonists on Osteoporosis

#### Effects of Glucagon-Like Peptide-1 Receptor Agonists on Diabetic Osteoporosis

Most patients with T2DM have normal bone density, but the risk of fracture is increased. The phenomenon is called the “bone fragility diabetes paradox,” indicating that other factors besides BMD may affect fracture risk. Therefore, the National Bone Health Alliance recommends that parameters of bone strength (such as changes of cortical pore and trabecular microstructure in bone) should be used to diagnose osteoporosis in T2DM. High-resolution computed tomography scans showed that postmenopausal women with T2DM had larger cortical pores than women without T2DM. Higher cortical porosity leads to lower bone strength and a higher incidence of fragility fractures in this population. In the course of clinical treatment, the impact of diabetes on bone health is often overlooked or underestimated. Both T2DM and T1DM are associated with a significantly increased risk of bone abnormalities and fractures. The mechanism of T1DM may be reduced BMD due to insufficient anabolic tone from insulin. There is a complex pathophysiological interaction between T2DM and osteoporosis. These factors include increased accumulation of advanced glycation end products (AGEs), chronic inflammation due to increased proinflammatory cytokines, and bone microvascular lesions with decreased vascular flow and increased bone fragility. Patients with chronic hyperglycemia may have a significant negative effect on bone mass. Because, in a long-term state of hyperglycemia, the non-enzymatic glycosylation of proteins, phospholipids and nucleic acids will lead to the continuous formation and accumulation of AGE. The excess AGEs could lead to non-enzymatic cross-linking of collagen, break the adhesion of osteoblasts to the extracellular matrix and leads to bone fragility (Mohsin et al., 2019). These changes in the extracellular matrix also reduced the alkaline phosphatase activity in mature osteoblasts and affected bone mineralization. AGEs receptor (RAGE) is expressed in human osteoclasts and stimulates the activation of nuclear factor Kappa-B in osteoclasts, thereby increasing the production of cytokines and reactive oxygen species. The continuous accumulation of pro-inflammatory cytokines and reactive oxygen species will break the balance between osteoclasts and osteoblasts, increase the differentiation of osteoclasts, and lead to bone loss. The excess AGEs increases chronic inflammation and bone resorption in diabetics. Therefore, we believe that patients with T1DM and T2DM are closely related to osteoporosis and fractures. The underlying mechanisms include changes in bone mechanical properties caused by non-enzymatic glycosylation, mineralization disorders, and bone micro-damages. At present, clinical treatment is mainly through restricting the patient’s diet and regulating blood sugar, strengthening daily exercise and supplementing calcium and vitamins, and using bone formation promoters and bone loss inhibitors for comprehensive treatment. Studies have found that GLP-1RAs and their analogs have the effect of alleviating diabetic osteoporosis (Mabilleau et al., 2018). The physiological role of GLP-1 mainly regulates glucose levels by stimulating the secretion of insulin, inhibiting the secretion of glucagon and regulating gastric emptying, thereby enhancing bone formation (Hare et al., 2009). ApoE^−/−^ mice are mainly used to study atherosclerosis, but ApoE^−/−^ mice can also be used to study osteoporosis. Osteoporosis is closely related to hyperlipidemia (Hjortnaes et al., 2010). A study investigate the effects of liraglutide on the advanced glycation end products (AGEs)-induced chronic diabetic complications of bone injury in ApoE^−/−^mice. The result showed that AGEs could increase bone resorption by reducing OC and increasing CTX but liraglutide could significantly decrease AGEs and parathyroid hormone (PTH) in ApoE^−/−^mice (Zhang et al., 2019a).

There is still a lack of clinical studies on the efficacy of GLP-1RAs in patients with osteoporosis. GLP-1RAs for the prevention of osteoporosis and fracture are controversial. A cohort study showed that there was no decreased risk of fracture with current use of GLP-1RA (sexenatide and liraglutide) compared to never-GLP-1RAs use. GLP-1RAS use did not significantly reduce the risk of osteoporotic fractures. A 52 weeks, controlled trial, investigated the efficacy of GLP-1RAs of liraglutide (1.2 and 1.8 mg/day) vs. glimepiride in T2DM. The results showed that at 52 weeks or 104 weeks, patients with liraglutide (1.8 or 1.2 mg/day) or glimepiride (8 mg/day) had no significant difference in average total bone mass change compared with baseline. In this 2 years prospective study, the researchers found that liraglutide monotherapy did not affect the patient’s total bone mineral density (Gilbert et al., 2016). But, a meta-analysis included 38 randomized controlled trials with 39,795 patients with T2DM. The result showed that the use of GLP-1RAs could significantly reduce the risk of fracture in patients, and the beneficial effect depends on the duration of treatment (Cheng et al., 2019). In our previous study, we analyzed 54 eligible random control trials treated with GLP-1RAs. The results also showed that exenatide treatment was likely to prevent fractures in treated T2DM patients compared with placebo or other anti-hyperglycemic drugs (Zhang et al., 2018). These results give us a direction that GLP-1RAs, currently used for diabetes, may be an alternative drug for the treatment of osteoporosis. Therefore, it is controversial whether GLP-1RAs therapy could prevent and treat diabetic osteoporosis and fragility fractures in clinical practice.

#### Effects of Glucagon-Like Peptide-1 Receptor Agonists on Senile Osteoporosis

Senile osteoporosis is primary osteoporosis, and its main characteristics are changes in bone microstructure, decreased bone density, and decreased bone strength. Senile osteoporosis has become a major global public health problem with an increasing number of fractures, disabilities and high socio-economic costs. Zhang et al. (2019b) evaluated the anti-osteoporosis effect of exendin-4 using a rat model of senescent osteoblast. The result found that Exendin-4 improved proliferation of senescent osteoblasts and ALP activity. PCR showed that Exendin-4 down-regulated the expression of senescence associated gene (p16, p21, p53) and up-regulated the expression of bone-related gene.

#### Effects of Glucagon-Like Peptide-1 Receptor Agonists on Glucocorticoid-Induced Osteoporosis

Glucocorticoid (GC) treatment is the main cause of secondary osteoporosis. The current drugs for treating and preventing GC-induced osteoporosis are insufficient. In our laboratory, we investigated the protective effect of geniposide (GLP-1RA) in dexamethasone-induced osteogenic inhibition. The results show that geniposide can promote ALP activity and mineralization. Furthermore, geniposide also significantly increased the expression of osteogenic genes of OPN, Runx2 and Osterix (Osx) in MC3T3-E1 cells treated with dexamethasone. GLP-1RA (geniposide) might be a potential therapeutic agent for GIOP (Xie et al., 2019). Yang et al. (2019) further verified our results by animal experiment. The result indicate that liraglutide could significantly improve BMD, bone microstructure, bone biomechanical markers, ALP, OC and decrease TRACP and serum c-terminal telopeptide of type 1 collagen (CTX-I), compared with the dexamethasone group. GLP-1RAs may improve BMD, bone strength and bone microstructure and reversed GIOP, primarily through slow down of bone resorption and promotion of bone formation and osteogenic differentiation.

#### Effects of Glucagon-Like Peptide-1 Receptor Agonists on Postmenopausal Osteoporosis

In one study, liraglutide was used to treat streptozotocin (STZ)-induced diabetes and/or bilateral ovariectomy (OVX) rats for 8 weeks. The results showed that liraglutide reduced serum CTX-1 levels and the number of osteoclasts in OVX and STZ + OVX rats. The result showed liraglutide significantly decreased serum CTX-1 level and osteoclast numbers in OVX and STZ + OVX rats and alleviated reduction of femoral BMD and destruction of bone microarchitecture in STZ and OVX induced rats. Liraglutide mainly inhibited osteoclast differentiation and alleviated bone deterioration and bone loss caused by STZ + OVX-induced rat models (Wen et al., 2018). Other study evaluated the impacts of liraglutide on bone mass in OVX-induced rats without diabetes, and the effect on the adipogenic and osteogenic differentiation of BMSCs. The results showed that liraglutide increased the expression of ALP, Col-1 and Runx2 mRNA, and decreased the expression of peroxisome proliferator-activated receptor γ (PPARγ) (Lu et al., 2015). Liraglutide has a bone protective effect even in non-diabetic osteoporotic OVX rats. Another study showed that GLP-1RA of liraglutide and exenatide can increase bone mass, structural parameters and connectivity in ovariectomized mice (Pereira et al., 2015). Montes Castillo et al. (2019) found that postprandial GLP-1 levels are related to osteoporosis risk in non-diabetic postmenopausal women. The result showed that GLP-1 are associated with reduced osteoporosis risk in the crude logistic regression analysis (*p* = 0.031) in non-diabetic postmenopausal women. These preliminary results indicate that GLP-1RAs have a potentially beneficial effect on bone metabolism in postmenopausal patients.

### Related Molecular Mechanisms of Glucagon-Like Peptide-1 Receptor Agonists in Osteoporosis

#### Glucagon-Like Peptide-1 Receptor/cAMP/PI3K/AKT Signal Pathway

**FIGURE 1:**
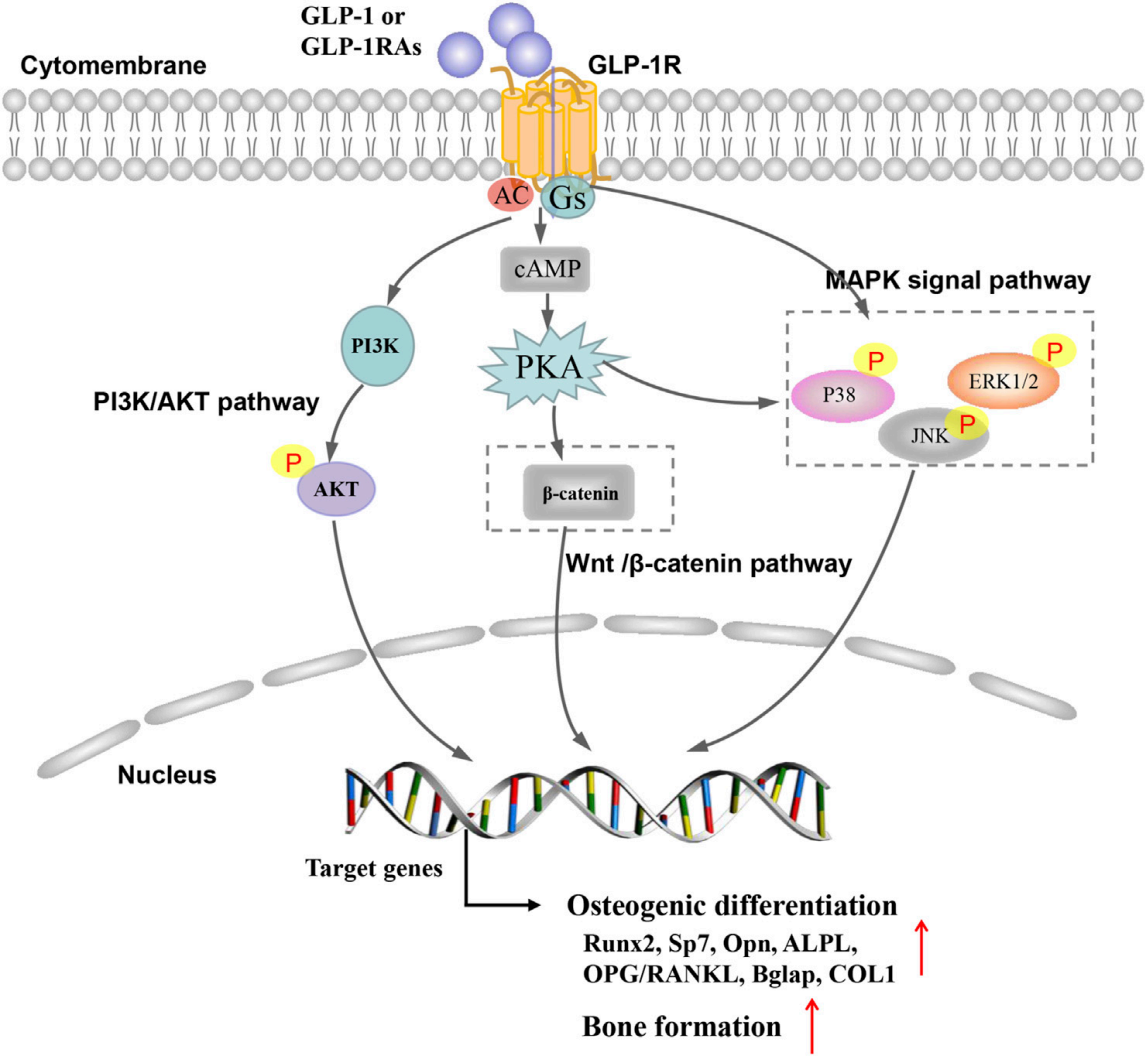
Possible mechanism of glucagon-like peptide 1 receptor agonist on bone metabolism in osteoporosis.

The PI3K/AKT signaling pathway plays an important role in regulating proliferation, osteoblast differentiation and osteoclast differentiation, apoptosis (Zhang et al., 2020; Ye et al., 2019). Studies have shown that GLP-1RA can regulate PI3K/AKT signaling pathway through GLP-1R to alleviate cell apoptosis (Xie et al., 2018; Ming-Yan et al., 2019). In addition, liraglutide could increase intracellular cAMP level and phosphorylation of AKT and the inhibitors LY294002, and GLP-1R siRNA could partially block the liraglutide-induced signaling activation and attenuated the facilitating effect of liraglutide. Therefore, liraglutide can activate PI3K/AKT and cAMP/PKA signals by binding to GLP-1 receptors, acting on osteoblasts, thereby promoting osteogenic differentiation and bone formation (Wu et al., 2017). Furthermore, the role of the GLP-1R/PI3K/AKT signaling pathway was also demonstrated in BMSC experiments. Activation of GLP-1R by exendin-4 promoted the osteogenic differentiation, increased bone formation and osteoblast number and inhibited BMSC adipogenic differentiation, but wortmannin (PI3K inhibitor), and GLP-1R sh-RNA partially blocked the exendin-4-induced PI3K/AKT signaling activation and attenuated the facilitating effect of exendin-4 in BMSCs (Meng et al., 2016) (Figure 1).

#### Glucagon-Like Peptide-1 Receptor/Mitogen-Activated Protein Kinase Signal Pathway

Mitogen-activated protein kinase (MAPK) belongs to silk/threonine protein kinase, mainly including extracellular signal-regulated kinase (Erk1/2), Jun N-terminal kinase (JNK) subfamily, p38 mitogen-activated protein kinase and ERK5 (Wang, 2007; Wagner and Nebreda, 2009; Arthur and Ley, 2013). The extracellular ligand play a role in regulating bone formation and skeletal metabolism, at least in part, through signal MAPK pathways (Greenblatt et al., 2010). Studies have shown that GLP-1RA regulate MAPK signaling pathway through GLP-1R to play the anti-diabetes, anti-tumor and anti-atherosclerosis effects (Nomiyama et al., 2014; Tang et al., 2019; Yue et al., 2019). Studies have found that GLP-1RA have similar effects in alleviating osteoporosis and preventing fractures (Nuche-Berenguer et al., 2010; Feng et al., 2016). Feng et al. (2016) found that exendin-4 increased the mRNA expression of ALP, COL1, OC, and Runx2 and Exendin-4 could up-regulate the phosphorylation of JNK, ERK1/2 and p38. But, the selective MAPK inhibitors of SB203580, SP600125 and PD98059 can block exendin-4-induced phosphorylation of JNK, ERK1/2 and p38. These findings demonstrate that exendin-4 may promote both the proliferation and differentiation of MC3T3-E1 by activating MAPK pathway. Previous research in our team found that GLP-1RA (geniposide) could significantly increase the mRNA and protein expression of OPN, Runx2, and Osterix in DEX-treated MC3T3-E1 cells by activating the ERK MAPK signaling pathway. In our previous studies, the GLP-1 receptor antagonist exendin 9–39 and ERK activation inhibitor U0126 could effectively block geniposide-induced ERK activation, thereby inhibiting the protective effect of geniposide (Xie et al., 2019). Other GLP-1RAs (liraglutide) also elevated phosphorylation of ERK with increased transcriptional activity and the inhibitors of PD98059 and GLP-1R siRNA partially blocked the liraglutide-induced ERK signaling activation (Wu et al., 2017). In short, the effects of GLP-1RAs were at least partially associated with activating MAPK signaling pathway *via* GLP-1R (Figure 1).

#### Wnt/**β**-catenin Signaling Pathway

Wnt/β-catenin pathway is a vital pathway regulating in regulating cell proliferation, apoptosis and tissue repair. The activation of Wnt/β-catenin pathway can promote the proliferation and differentiation of mesenchymal stem cells and osteoblasts, increase cell activity and promote osteogenic differentiation (Deng et al., 2019; Molagoda et al., 2019). Activation of GLP-1R by exendin-4 could promot the osteogenic differentiation and inhibite adipogenic differentiation through regulating PKA/β-catenin signaling pathway (Meng et al., 2016). Liraglutide could increase intracellular cAMP levels and β-catenin levels, while increasing the nuclear β-catenin content and transcriptional activity. β-catenin siRNA partially blocked the signal activation induced by liraglutide and weakened the promoting effect of liraglutide on MC3T3-E1 cells (Wu et al., 2017) (Figure 1).

#### OPG/RANKL Signaling Pathway

The OPG/RANKL signaling system is critical in the pathogenesis of bone related diseases caused by increased bone resorption (Hofbauer and Schoppet, 2004; Yang et al., 2020). Nuche-Berenguer et al. (2009) evaluated bone metabolic markers in streptozotocin-induced T2DM rat models. The result observed that the ratio of OPG/RANKL in T2DM was 0.81 and that this value rose to a height of 1.25 after GLP-1 treatment. GLP-1RA of Exendin-4 mainly inhibited bone resorption by regulating OPG/RANKL ratio, and increases the expression of osteogenic related genes OC, COL1, Runx2, and ALP to promote bone formation (Ma et al., 2013). In addition, this study detected OPG and RANKL mRNA expression and serum concentration. Liraglutide could significantly increase the OPG/RANKL ratio, indicating that liraglutide could inhibit the formation of osteoclasts.

## Conclusions and Expectations

At present, there are evidences that GLP-1RAs and their analogs can enhance bone density and improve bone quality. GLP-1RAs and their analogs may play an anti-osteoporosis role by promoting bone formation and inhibiting bone absorption. However, the specific impact of GLP-1RA on fracture risk and osteoporosis has not been unambiguously defined. Some studies have proven that GLP-1RAs have favorable anabolic effects on skeletal metabolism, but underlying molecular mechanism remains to be elucidated. The initial results of GLP-1RAs effects on bone metabolism and anti-osteoporosis sound promising and meaningful, but they should be interpreted with caution and in the context of the trials.

## References

[B1] Ali M. K. Bullard K. M. Saydah S. Imperatore G. Gregg E. W. (2018). Cardiovascular and Renal Burdens of Prediabetes in the USA: Analysis of Data from Serial Cross-Sectional Surveys, 1988–2014. Lancet Diabetes Endocrinol. 6 (5), 392–403. 10.1016/S2213-8587(18)30027-5 29500121 PMC6615033

[B2] Aroda V. R. (2018). A Review of GLP-1 Receptor Agonists: Evolution and Advancement, through the Lens of Randomised Controlled Trials. Diabetes Obes. Metab. 20 (Suppl. 1), 22–33. 10.1111/dom.13162 29364586

[B3] Arthur J. S. C. Ley S. C. (2013). Mitogen-activated Protein Kinases in Innate Immunity. Nat. Rev. Immunol. 13 (9), 679–692. 10.1038/nri3495 23954936

[B4] Cheng L. Hu Y. Li Y. Y. Cao X. Bai N. Lu T. T. (2019). Glucagon‐like Peptide‐1 Receptor Agonists and Risk of Bone Fracture in Patients with Type 2 Diabetes: A Meta‐analysis of Randomized Controlled Trials. Diabetes Metab. Res. Rev. 35 (7), e3168. 10.1002/dmrr.3168 30974033

[B5] Chun J. H. Butts A. (2020). Long-acting GLP-1RAs. JAAPA 33 (8), 3–18. 10.1097/01.JAA.0000669456.13763.bd 32740121

[B6] Deng Q. Li P. Che M. Liu J. Biswas S. Ma G. (2019). Activation of Hedgehog Signaling in Mesenchymal Stem Cells Induces Cartilage and Bone Tumor Formation via Wnt/β-Catenin. eLife 8, e50208. 10.7554/eLife.50208 31482846 PMC6764825

[B7] Feng Y. Su L. Zhong X. Guohong W. Xiao H. Li Y. (2016). Exendin-4 Promotes Proliferation and Differentiation of MC3T3-E1 Osteoblasts by MAPKs Activation. J. Mol. Endocrinol. 56 (3), 189–199. 10.1530/JME-15-0264 26647389

[B8] Gilbert M. P. Marre M. Holst J. J. Garber A. Baeres F. M. Thomsen H. (2016). Comparison of the Long-Term Effects of Liraglutide and Glimepiride Monotherapy on Bone Mineral Density in Patients with Type 2 Diabetes. Endocr. Pract. 22 (4), 406–411. 10.4158/EP15758.OR 26574791

[B9] Greenblatt M. B. Shim J.-H. Zou W. Sitara D. Schweitzer M. Hu D. (2010). The P38 MAPK Pathway Is Essential for Skeletogenesis and Bone Homeostasis in Mice. J. Clin. Invest. 120 (7), 2457–2473. 10.1172/JCI42285 20551513 PMC2898605

[B10] Hare K. J. Knop F. K. Asmar M. Madsbad S. Deacon C. F. Holst J. J. (2009). Preserved Inhibitory Potency of GLP-1 on Glucagon Secretion in Type 2 Diabetes Mellitus. J. Clin. Endocrinol. Metab. 94 (12), 4679–4687. 10.1210/jc.2009-0921 19837930

[B11] Hjortnaes J. Butcher J. Figueiredo J.-L. Riccio M. Kohler R. H. Kozloff K. M. (2010). Arterial and Aortic Valve Calcification Inversely Correlates with Osteoporotic Bone Remodelling: a Role for Inflammation. Eur. Heart J. 31 (16), 1975–1984. 10.1093/eurheartj/ehq237 20601388 PMC2921509

[B12] Hofbauer L. C. Schoppet M. (2004). Clinical Implications of the osteoprotegerin/RANKL/RANK System for Bone and Vascular Diseases. JAMA 292 (4), 490–495. 10.1001/jama.292.4.490 15280347

[B13] Lindamood C. A. Taylor J. R. (2015). Emerging New Therapies for the Treatment of Type 2 Diabetes Mellitus: Glucagon-like Peptide-1 Receptor Agonists. Clin. Ther. 37 (3), 483–493. 10.1016/j.clinthera.2015.01.003 25659912

[B14] Lu N. Sun H. Yu J. Wang X. Liu D. Zhao L. (2015). Glucagon-like Peptide-1 Receptor Agonist Liraglutide Has Anabolic Bone Effects in Ovariectomized Rats without Diabetes. PLoS One 10 (7), e0132744. 10.1371/journal.pone.0132744 26177280 PMC4503456

[B15] Ma X. Meng J. Jia M. Bi L. Zhou Y. Wang Y. (2013). Exendin-4, a Glucagon-like Peptide-1 Receptor Agonist, Prevents Osteopenia by Promoting Bone Formation and Suppressing Bone Resorption in Aged Ovariectomized Rats. J. Bone Miner Res. 28 (7), 1641–1652. 10.1002/jbmr.1898 23427056

[B16] Mabilleau G. Pereira M. Chenu C. (2018). Novel Skeletal Effects of Glucagon-like Peptide-1 (GLP-1) Receptor Agonists. J. Endocrinol. 236 (1), R29–R42. 10.1530/JOE-17-0278 28855317

[B17] Meier J. J. (2012). GLP-1 Receptor Agonists for Individualized Treatment of Type 2 Diabetes Mellitus. Nat. Rev. Endocrinol. 8 (12), 728–742. 10.1038/nrendo.2012.140 22945360

[B18] Meng J. Ma X. Wang N. Jia M. Bi L. Wang Y. (2016). Activation of GLP-1 Receptor Promotes Bone Marrow Stromal Cell Osteogenic Differentiation through β-Catenin. Stem Cel. Rep. 6 (4), 579–591. 10.1016/j.stemcr.2016.02.002 PMC4834036 26947974

[B19] Ming-Yan Y. Jing Z. Shu-Qin G. Xiao-Liang B. Zhi-Hong L. Xue Z. (2019). Liraglutide Inhibits the Apoptosis of Human Nucleus Pulposus Cells Induced by High Glucose through PI3K/Akt/caspase-3 Signaling Pathway. Biosci. Rep 39 (8), BSR20190109. 10.1042/BSR20190109 31383790 PMC6702359

[B20] Mohler M. L. He Y. Wu Z. Hwang D. J. Miller D. D. (2009). Recent and Emerging Anti-diabetes Targets. Med. Res. Rev. 29 (1), 125–195. 10.1002/med.20142 18855890

[B21] Mohsin S. Baniyas M. M. Aldarmaki R. S. Tekes K. Kalász H. Adeghate E. A. (2019). An Update on Therapies for the Treatment of Diabetes-Induced Osteoporosis. Expert Opin. Biol. Ther. 19 (9), 937–948. 10.1080/14712598.2019.1618266 31079501

[B22] Molagoda I. M. N. Karunarathne W. A. H. M. Choi Y. H. Park E. K. Jeon Y.-J. Lee B.-J. (2019). Fermented Oyster Extract Promotes Osteoblast Differentiation by Activating the Wnt/β-Catenin Signaling Pathway, Leading to Bone Formation. Biomolecules 9 (11), 711. 10.3390/biom9110711 PMC6920898 31698882

[B23] Montes Castillo M. C. Martínez Ramírez M. J. Soriano Arroyo R. Prieto Gomez I. Segarra Robles A. B. Garrido-Martínez M. (2019). Glucagon-like Peptide 1 and Glucagon-like Peptide 2 in Relation to Osteoporosis in Non-diabetic Postmenopausal Women. Sci. Rep. 9 (1), 13651. 10.1038/s41598-019-50117-z 31541189 PMC6754449

[B24] Napoli N. Chandran M. Chandran M. Pierroz D. D. Abrahamsen B. Schwartz A. V. (2017). Mechanisms of Diabetes Mellitus-Induced Bone Fragility. Nat. Rev. Endocrinol. 13 (4), 208–219. 10.1038/nrendo.2016.153 27658727

[B25] Nomiyama T. Kawanami T. Irie S. Hamaguchi Y. Terawaki Y. Murase K. (2014). Exendin-4, a GLP-1 Receptor Agonist, Attenuates Prostate Cancer Growth. Diabetes 63 (11), 3891–3905. 10.2337/db13-1169 24879833

[B26] Nuche-Berenguer B. Moreno P. Esbrit P. Dapía S. Caeiro J. R. Cancelas J. (2009). Effect of GLP-1 Treatment on Bone Turnover in normal, Type 2 Diabetic, and Insulin-Resistant States. Calcif Tissue Int. 84 (6), 453–461. 10.1007/s00223-009-9220-3 19219381

[B27] Nuche-Berenguer B. Portal-Núñez S. Moreno P. González N. Acitores A. López-Herradón A. (2010). Presence of a Functional Receptor for GLP-1 in Osteoblastic Cells, Independent of the cAMP-Linked GLP-1 Receptor. J. Cel. Physiol. 225 (2), 585–592. 10.1002/jcp.22243 20506394

[B28] Pereira M. Jeyabalan J. Jørgensen C. S. Hopkinson M. Al-Jazzar A. Roux J. P. (2015). Chronic Administration of Glucagon-like Peptide-1 Receptor Agonists Improves Trabecular Bone Mass and Architecture in Ovariectomised Mice. Bone 81, 459–467. 10.1016/j.bone.2015.08.006 26314515

[B29] Rawshani A. Rawshani A. Franzén S. Sattar N. Eliasson B. Svensson A.-M. (2018). Risk Factors, Mortality, and Cardiovascular Outcomes in Patients with Type 2 Diabetes. N. Engl. J. Med. 379 (7), 633–644. 10.1056/NEJMoa1800256 30110583

[B30] Schwartz A. V. Vittinghoff E. Bauer D. C. Hillier T. A. Strotmeyer E. S. Ensrud K. E. (2011). Association of BMD and FRAX Score with Risk of Fracture in Older Adults with Type 2 Diabetes. JAMA 305 (21), 2184–2192. 10.1001/jama.2011.715 21632482 PMC3287389

[B31] Sharma D. Verma S. Vaidya S. Kalia K. Tiwari V. (2018). Recent Updates on GLP-1 Agonists: Current Advancements & Challenges. Biomed. Pharmacother. 108, 952–962. 10.1016/j.biopha.2018.08.088 30372907

[B32] Tang S.-t. Tang H.-q. Su H. Wang Y. Zhou Q. Zhang Q. (2019). Glucagon-like Peptide-1 Attenuates Endothelial Barrier Injury in Diabetes via cAMP/PKA Mediated Down-Regulation of MLC Phosphorylation. Biomed. Pharmacother. 113, 108667. 10.1016/j.biopha.2019.108667 30852419

[B33] Wagner E. F. Nebreda Á. R. (2009). Signal Integration by JNK and P38 MAPK Pathways in Cancer Development. Nat. Rev. Cancer 9 (8), 537–549. 10.1038/nrc2694 19629069

[B34] Wang Y. (2007). Mitogen-activated Protein Kinases in Heart Development and Diseases. Circulation 116 (12), 1413–1423. 10.1161/CIRCULATIONAHA.106.679589 17875982 PMC3808829

[B35] Wen B. Zhao L. Zhao H. Wang X. (2018). Liraglutide Exerts a Bone protective Effect in Ovariectomized Rats with Streptozotocin induced Diabetes by Inhibiting Osteoclastogenesis. Exp. Ther. Med. 15 (6), 5077–5083. 10.3892/etm.2018.6043 29805533 PMC5958780

[B36] Wu X. Li S. Xue P. Li Y. (2017). Liraglutide, a Glucagon-like Peptide-1 Receptor Agonist, Facilitates Osteogenic Proliferation and Differentiation in MC3T3-E1 Cells through Phosphoinositide 3-kinase (PI3K)/protein Kinase B (AKT), Extracellular Signal-Related Kinase (ERK)1/2, and cAMP/protein Kinase A (PKA) Signaling Pathways Involving β-catenin. Exp. Cel Res. 360 (2), 281–291. 10.1016/j.yexcr.2017.09.018 28919123

[B37] Xie B. Wu J. Li Y. Wu X. Zeng Z. Zhou C. (2019). Geniposide Alleviates Glucocorticoid-Induced Inhibition of Osteogenic Differentiation in MC3T3-E1 Cells by ERK Pathway. Front. Pharmacol. 10, 411. 10.3389/fphar.2019.00411 31057410 PMC6482204

[B38] Xie Z. Enkhjargal B. Wu L. Zhou K. Sun C. Hu X. (2018). Exendin-4 Attenuates Neuronal Death via GLP-1R/PI3K/Akt Pathway in Early Brain Injury after Subarachnoid Hemorrhage in Rats. Neuropharmacology 128, 142–151. 10.1016/j.neuropharm.2017.09.040 28986282 PMC5714662

[B39] Xu G. Liu B. Sun Y. Du Y. Snetselaar L. G. Hu F. B. (2018). Prevalence of Diagnosed Type 1 and Type 2 Diabetes Among US Adults in 2016 and 2017: Population Based Study. BMJ 362, k1497. 10.1136/bmj.k1497 30181166 PMC6122253

[B40] Yang B. Li S. Chen Z. Feng F. He L. Liu B. (2020). Amyloid β Peptide Promotes Bone Formation by Regulating Wnt/β‐catenin Signaling and the OPG/RANKL/RANK System. FASEB j. 34 (3), 3583–3593. 10.1096/fj.201901550R10.1096/fj.201901550R 31944393

[B41] Yang L. Yang J. Pan T. Zhong X. (2019). Liraglutide Increases Bone Formation and Inhibits Bone Resorption in Rats with Glucocorticoid-Induced Osteoporosis. J. Endocrinol. Invest. 42 (9), 1125–1131. 10.1007/s40618-019-01034-5 30955181

[B42] Ye C. Zhang W. Hang K. Chen M. Hou W. Chen J. (2019). Extracellular IL-37 Promotes Osteogenic Differentiation of Human Bone Marrow Mesenchymal Stem Cells via Activation of the PI3K/AKT Signaling Pathway. Cell Death Dis 10 (10), 753. 10.1038/s41419-019-1904-7 31582734 PMC6776644

[B43] Yue W. Li Y. Ou D. Yang Q. (2019). The GLP‐1 Receptor Agonist Liraglutide Protects against Oxidized LDL‐induced Endothelial Inflammation and Dysfunction via KLF2. Iubmb Life 71 (9), 1347–1354. 10.1002/iub.2046 30969479

[B44] Zhang L. Li P. Tang Z. Dou Q. Feng B. (2019a). Effects of GLP-1 Receptor Analogue Liraglutide and DPP-4 Inhibitor Vildagliptin on the Bone Metabolism in ApoE−/− Mice. Ann. Transl. Med. 7 (16), 369. 10.21037/atm.2019.06.74 31555683 PMC6736830

[B45] Zhang M. Xie Y. Zhou Y. Chen X. Xin Z. An J. (2019b). Exendin-4 Enhances Proliferation of Senescent Osteoblasts through Activation of the IGF-1/IGF-1R Signaling Pathway. Biochem. Biophysical Res. Commun. 516 (1), 300–306. 10.1016/j.bbrc.2019.06.112 31256933

[B46] Zhang Y. Cao X. Li P. Fan Y. Zhang L. Li W. (2020). PSMC6 Promotes Osteoblast Apoptosis through Inhibiting PI3K/AKT Signaling Pathway Activation in Ovariectomy‐induced Osteoporosis Mouse Model. J. Cel. Physiol. 235, 5511–5524. 10.1002/jcp.2926110.1002/jcp.29261 32017075

[B47] Zhang Y. S. Weng W. Y. Xie B. C. Meng Y. Hao Y. H. Liang Y. M. (2018). Glucagon-like Peptide-1 Receptor Agonists and Fracture Risk: a Network Meta-Analysis of Randomized Clinical Trials. Osteoporos. Int. 29 (12), 2639–2644. 10.1007/s00198-018-4649-8 30083774

